# PROTOCOL: Effectiveness of interpersonal psychotherapy in comparison to other psychological and pharmacological interventions for reducing depressive symptoms in women diagnosed with postpartum depression in low and middle‐income countries: A systematic review

**DOI:** 10.1002/cl2.1074

**Published:** 2020-02-25

**Authors:** Harmeet Kaur Kang, Denny John, Bandana Bisht, Manmeet Kaur, Obrey Alexis, Aaron Worsley

**Affiliations:** ^1^ Chitkara School of Health Sciences Chitkara University Punjab India; ^2^ Campbell Collaboration New Delhi India; ^3^ Chitkara University College of Nursing Chitkara University Himachal Pradesh India; ^4^ Faculty of Health and Life Sciences Oxford Brookes University Swindon UK

## Abstract

Postpartum Depression (PPD) is highly prevalent among women in low and middle income countries (LMICs). World Heath Organization has recognised interpersonal Psychotherapy (IPT) as the first line treatment for the postpartum depression. The primary aim of this review is to evaluate the effectiveness of IPT alone or in combination with pharmacotherapy or other psychosocial therapies for treating depressive symptoms in women with postpartum depression. The generated evidence from this review will help to inform policies in relation to the treatment of postpartum depression in LMICs.

## 1 BACKGROUND

Depression is the most common mental health condition affecting perinatal women and mothers (Hanlon, 2013). It is estimated that 15–85% of women experience postpartum blues within 10 days of giving birth which is a subsequent risk factor for postpartum depression (Pearlstein, Howard, Salisbury, & Zlotnick, 2009). Evidence suggests that one out of seven women suffer from postpartum depression (PPD; American Psychiatric Association, 2013). In psychiatric nomenclature, postpartum depression is defined as a major depressive disorder with a specifier of postpartum onset. According to the Diagnostic Statistical Manual‐5 (DSM‐5; American Psychiatric Association, 2013), PPD is defined as, ‘a major depression episode during pregnancy or within 4 weeks of delivery’. Whereas, the International Classification of Diseases‐10 (ICD‐10) guidelines state that the onset of postpartum depression is considered to be within 6 weeks after delivery (WHO, 2016a). This condition is twice more prevalent in low middle income countries (LMICs) as compared to high income countries (Gajaria and Ravindran, 2018)

PPD is known to affect mothers regardless of the fact that they may have had an easy or problematic pregnancy. It has been reported that approximately 19% of mothers from LAMICs will experience significant depressive symptoms during the first 3 months after giving birth. Postpartum depression in LAMICs is highly prevalent affecting one out of five women (Gelaye, Rondon, Araya, & Williams Michelle, 2016). The prevalence rate of PPD in LMICs is 31.8% which is higher than developed countries, that is, 21.5%. Futhermore, the prevalence of PPD in LMICs among rural women is higher than the rural women of developed countries (Fisher et al., 2012).

Women experiencing postpartum depression generally may exhibit symptoms such as depressed mood, anxiety, feelings of guilt or inadequacy, inability to cope, loss of control, intrusive presence of compulsive thoughts, irrational fears and despair (American Psychiatric Association, 2013). Moreover, postpartum depression can predispose to chronic or recurrent depression, which may affect the mother ‐ infant relationship and child's growth and development (Miniati et al., 2014). As per the literature, mother's with previous or recurrent depression are more prone to have the positive signs of depression even in postpartum period because of the alteration of the chemical balance in channels called as neurotransmitters (Hanlon, 2013). Mothers suffering from PPD do not enjoy their baby and often believe that they are bad parents. There could be thoughts related to harming self or the baby CMHA 2014. Further, children of mothers with postpartum depression have greater cognitive, behavioral and interpersonal problems compared with children of non‐depressed mothers (Slomian, Honvo, Emonts, Reginster, & BruyÃ¨re, 2019).

Pregnancy is a sensitive period where various bio psychosocial variants that influence the psychology of women (Slomian, Honvo, Emonts, Reginster, & BruyÃ¨re, 2019). The significant predictors causing PPD include biological factors. Studies demonstrated that women who are younger were more likely to experience postpartum depression (Ghaedrahmati, Kazemi, Kheirabadi, Ebrahimi, & Bahrami, 2017). Additionally, women with glucose metabolism disorders were at risk of developing postpartum depression (Huang et al., 2015). Moreover changes in role transitions and interpersonal factors (e.g., partnership satisfaction, social support) during or after pregnancy (Martini, 2015; Yuksel & Aydin, 2015). Not only are these factors impacting on the mental health and wellbeing of women, several social factors such as unmarried mothers and unplanned pregnancy, perceived lack of support from partners, parents, relatives and friends during the postpartum period and impaired interpersonal relationships between women with postpartum depression and their spouses (Chien, Tai, & Yeh, 2012; Ghaedrahmati, Kazemi, Kheirabadi, Ebrahimi, & Bahrami, 2017; Gurber, Baumeler, Grob, Surbek, & Stadlmayr, 2017; Ludermir, Lewis, Valongueiro, de Araujo, & Araya, 2010) have been shown to contribute towards PPD among women in both LAMICs and high income countries. Moreover, mothers with lower socioeconomic status and the primigravida mothers often experiences more incidence related to postpartum depression. The pertaining reasons for postpartum mothers residing in LMICs often experience financial burden, lack of family support, thus resulting into poor marital relationships and hence causing postpartum depression (Bener, Gerber, & Sheikh, 2012). People living in poverty in LMICs usually do not have access to health services, good nutrition or clean environment. They also face challenges of overcrowding and poor housing conditions which further adds layers of stress during and after pregnancy (Coast, Leone, Hirose, & Jones, 2012).

There are no viable guidelines in low‐ and middle‐income countries for treating postpartum depression. Hence WHO suggests integrating mental health services with primary care so as to reduce the treatment gap for LAMICs. The usual treatment for PPD involves psychotherapy and antidepressants. Due to concerns of infant exposure to antidepressants, the preferred choice of treatment for mothers is psychotherapy (Stuart & O'Hara, 2012). Interpersonal psychotherapy (IPT) has been suggested as the most relevant to postpartum depression as IPT targets the specific interpersonal problems experienced by women in the postpartum period (Gao, Chan, Li, Chen, & Hao, 2010; Stuart & O'Hara, 2012). IPT is based on the premise that interpersonal problems encountered during pregnancy along with various hormonal changes results into possible symptoms of depression. Treatment modalities delivered by mental health care professionals in low and high middle income countries for mothers with PPD can help achieve the positivity in terms of better therapeutic outcomes along with psychosocial improvement. Many studies have demonstrated the efficacy of IPT in depression over other psychological treatments (Bolton et al., 2003; Mufson et al., 2004; Markowitz & Weissman, 2012; Beeber et al., 2013; Posmontier, Neugebauer, Stuart, Chittams, & Shaughnessy, 2016). Various organizations such as the American Psychiatric Association (2013) and the National Institute for Health and Clinical Excellence: Guidance (2014), have recognized IPT as an efficacious psychological therapy. Despite the growing number of empirical studies on postpartum depression, there is a lack of robust systematic evidence on efficacy of IPT to treat PPD among LMICs. Therefore, this systematic review will determine the usefulness of IPT to treat PPD among mothers in LMICs.

### 1.1 Description of the condition

Post partum depression is a non‐psychotic depressive episode in which a women experiences symptoms of sadness, anxiety, loneliness, anhedonia, despondency, emotional lability, crying spells, anger or rage during or within 6 months after childbirth. It is different from postpartum blues as the latter has similar symptoms but usually resolves in few days without any treatment. Post partum depression if left unattended can have adverse effects. It not just challenges the way women has to take care of her offspring but also greatly affects her own daily self care activities (NIMH, 2013). The mother with depression has increased likelihood of committing suicide. Prolonged depression in mother can hamper mother‐infant bonding, breastfeeding and infant care (WHO 2008). WHO estimates the prevalence rate of PPD to be 10% which reach up to 15–20% in developing countries (WHO 2008). The common determinants of PPD in LMICs is low socio‐economic status, lack of social support, resentment due to the sex of the baby resulting into poor relationship with partner or mother‐in‐law (Shidhaye & Giri, 2014). The treatment of PPD involves pharmacotherapy, psychotherapy and sometimes both.

### 1.2 Description of the intervention

IPT is a brief time‐limited psychotherapy in which the focus of the treatment is current concerns and improving interpersonal relationships (Markowitz & Weissman, 2004). IPT has proven efficacious in the treatment of affective disorders, anxiety disorders, eating disorders, post traumatic stress disorder and depression (Omay & Stuart, 2013). IPT has wide adaptability. It can be provided in all settings, including hospitals and community settings. It can be given individually as well as in group (WHO, 2013). IPT is based on the principle that relationships and life events impact on mood. It was developed by Gerald Klerman and Myrna Weissman for major depression in the 1970s and has since been adapted for other mental disorders. The four problem areas explored in IPT (Klerman & Weissman, 1993; Omay & Stuart, 2013; WHO, 2016a) are grief, role disputes, life changes/role transition, and loneliness/social isolation/Interpersonal deficits. Phases of IPT include initial, middle and termination phase.

- Initial phase: This includes 1–3 initial sessions. The important tasks of this phase are taking the complete history of the client and building rapport. The therapist makes the diagnosis during this phase by using any standard tool for depression. Out of four problematic areas, both therapist and the client mutually decide to pick one or two areas which are of concern and will be addressed in subsequent sessions.
  ○ Grief: In this problem area, the therapist works on complicated bereavement and helps the client to compete normal cycle of grieving.
  ○ Role dispute: This problem area is chosen in case of unsatisfied interpersonal relationships and non‐reciprocal role expectations between two persons.
  ○ Role transitions: This problem area is chosen when there are major role transitions or role changes such as a new job, new relationship or change in socio‐economic status or health etc. Adaptive coping strategies are encouraged in the client.
  ○ Interpersonal deficits: This area is chosen when no other interpersonal problem area is identified. Often, there is a long history of impoverished interpersonal relationships.

- Middle phase: The middle phase is conducted from 4th to 13th sessions. As suggested by Klerman,one of the interpersonal problematic area is worked upon by assessing mal‐adaptive responses in each problem area (Klerman & Weissman, 1993).
- Termination phase: The final phase last from 14 to 16 sessions. Therapist and the patient review their treatment course and future treatment needs (Markowitz & Weissman, 2004).

On the basis of duration of IPT, it can be of two types:

1. Standard IPT: The originally developed IPT has a duration of 12–16 sessions
2. Brief IPT: Brief IPT has only eight sessions. It was developed in the paucity of evidence linking increased duration to better treatment outcomes. It helps the clients to take benefit of IPT in fewer sessions in case they have some constraints (Swartz, Grote, & Graham, 2014).

**Table:** 

DOMAIN	IPT	IPT‐B
Number of sessions	12‐16	8
Initial Phase	3	2
Middle Phase	7‐11	5
Termination	2‐3	1

IPT is a flexible therapy. It can be given in various formats such as:

1. Individual IPT
2. Group IPT
3. Couples IPT
4. Telephone IPT (Markowitz & Weissman, 2004)

Settings for IPT:

1. Health care settings
2. Community settings (Markowitz & Weissman, 2004)

IPT can be delivered by the following personnel:

1. Specialist providers (trained in IPT) (WHO, 2016b)
  • Psychiatrists
  • Psychologists
  • Social workers
  • Mental health nurses

2. Non‐specialist providers who have received training in IPT (WHO, 2016a)
  • Community workers
  • General physicians
  • Nurses
  • Graduates with social or behavioral science degrees

### 1.3 How the intervention might work

**Figure 1:**
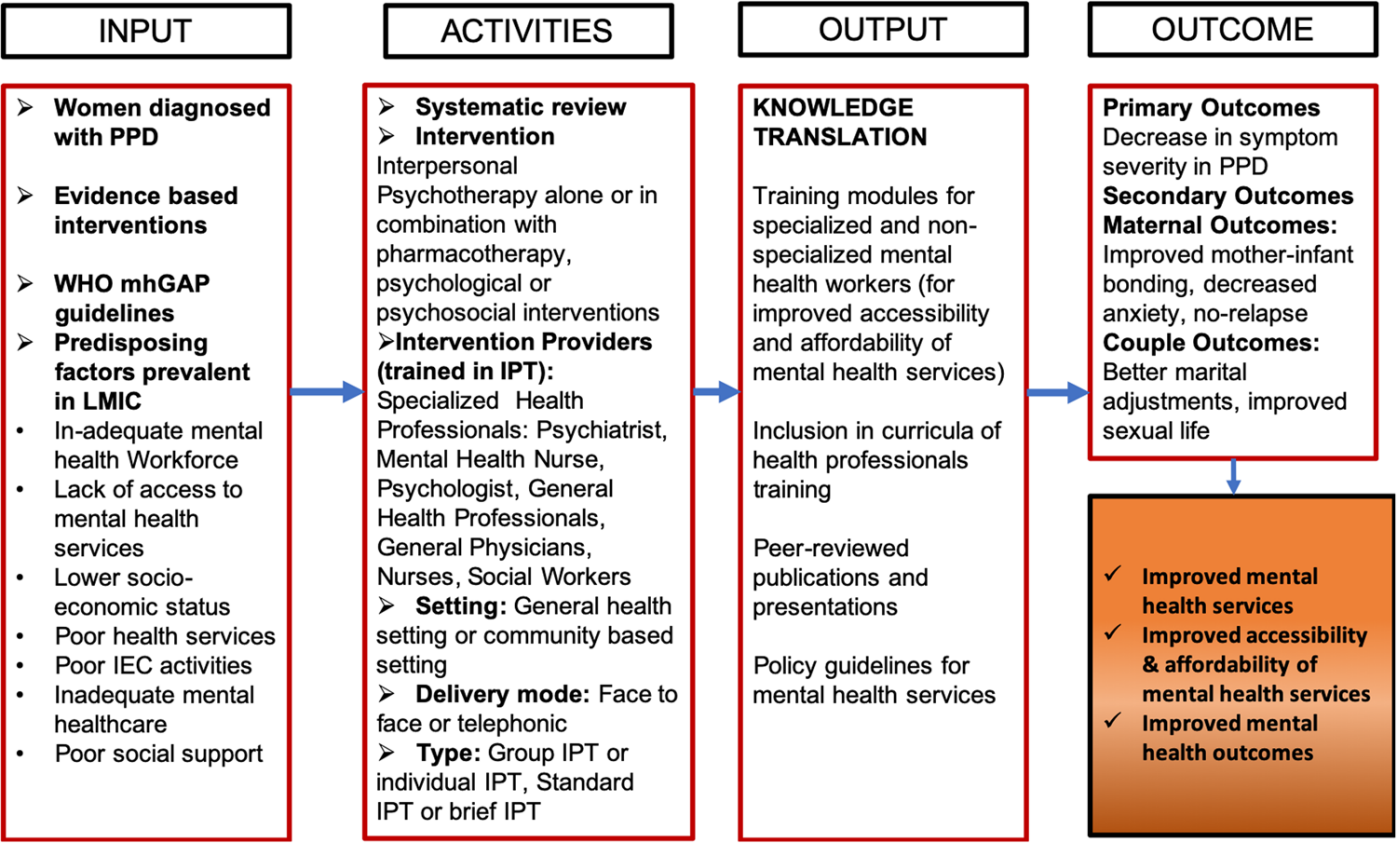
Logic Model concerning the effcetiveness of Interpersonal psychotherapy to improve postpartum depression outcomes and improved mental health services in LMICs (Von Bertalanffy, 1971)

Psychotherapy is a better treatment than pharmacotherapy for depression as there are fewer relapses and lesser premature termination of treatment (Hunsley, Elliott, & Therrien, 2013). Emerging evidence suggests better treatment adherence & decreased suicides if psychotherapy is added along with medication for the treatment of depression (Hunsley, Elliott, & Therrien, 2013). Comparison of various types of psychotherapies like cognitive behavior therapy (CBT), cognitive therapy, behavior therapy, play therapy, psychodynamic therapy, family therapy, problem solving therapy, supportive therapy and IPT have been examined in a number of clinical trials (Stamou, García‐Palacios, & Botella, 2018). CBT and IPT are more efficacious in treating depression as compared to other therapies (Cuijpers, 2016). Though IPT and CBT have equal or nearly equal effectiveness (Cuijpers, 2016; Power & Freeman, 2012), IPT has some added advantages over CBT (WHO, 2012). IPT can be given by non‐ specialized trained persons (Van Ginneken et al., 2013; WHO, 2016a). It can also be delivered telephonically (Bleiberg & Markowitz, 2005; O'Hara, Stuart, Gorman, & Wenzel, 2000). Unlike other therapies, the flexible approach of IPT increases its scope in terms of accessibility and affordability to a larger population. That is why, WHO has recommended IPT as the first line treatment for depression for pregnant and breastfeeding mothers with moderate‐severe depressive disorder (WHO, 2016b) (Figure 1).

The logic model has been adapted from Ludwig's systems model (Von Bertalanffy, 1971), where different factors influencing the outcome of treatment strategy (IPT) on postpartum depression are identified in LMICs. Potential influencing factors such as existing literature and evidence, WHO guidelines and predisposing factors in LMICs for the occurrence of PPD are presented in Input. The intervention for the treatment of PPD could be IPT alone or IPT with pharmacotherapy or IPT in combination with other psychological/psychosocial interventions in general health settings or community based settings. Mode of delivery could be face to face or telephonic conversation. IPT can be provided by Specialized health workers such as Psychiatrist, Psychologist or Mental health nurses or non‐specialized general health workers such as General physicians, Nurses and Social workers who are trained in IPT. IPT can be given in groups or individually. When 6–10 members are part of the therapy at one time, it is called Group‐IPT. The activities also include differentiation on the basis of duration of the therapy, that is, Standard IPT which of 12–16 weeks and Brief IPT, that is, of 8 weeks. The primary outcome of giving IPT would be decrease in symptoms severity among the mothers suffering from PPD. The secondary outcomes can fall under maternal outcomes and couple outcomes. Maternal outcomes can be measured in the form of improved mother‐infant bonding, decreased anxiety of the mother and no future relapse. IPT can lead to better couple outcomes in relation to better marital adjustments and improved sexual life. Hence providing IPT can lead to improved mental health services and mental health outcomes. It also impacts the community at large for providing accessible and affordable mental health treatment.

### 1.4 Why it is important to do this review

PPD in LMICs is under‐acknowledged and under‐researched (Coast, Leone, Hirose, & Jones, 2012). This review is important as previous reviews have not focused primarily on IPT especially in LMICs. Moreover, lack of resources in health care facilities and lags of treatment gaps are the major reasons for considering the treatment modalities especially in LMICs (Rathod et al., 2017. Few of the previous reviews and meta‐analysis have evaluated the effectiveness of all psychosocial and psychological treatments on postpartum depression (Dennis, 2005; O'Connor, Rossom, Henninger, Groom, & Burda, 2016; Sockol, Epperson, & Barber, 2011). These reviews had very few trials for the effectiveness of IPT on PPD, as a result no conclusive evidence was reported regarding the effectiveness of IPT on PPD. Existing reviews have focused on depressive symptoms reduction only (Dennis, 2005; Sockol, Epperson, & Barber, 2011). They did not study the efficacy of the intervention such as mode of IPT, duration of IPT (number of sessions) and combination of IPT and pharmacotherapy. Only one systematic review was conducted which specifically examined the effectiveness of IPT on PPD (Miniati et al., 2014). Other related outcomes such as maternal‐infant relationship, social adjustment, social support, couple and infant outcomes and harmful effects of IPT were not assessed. Further, another review studied the effects of dyadic relationship focused interventions on the mother‐infant relationships and child outcomes, but did not provide any substantial evidence as to whether improvement in mother ‐infant relationship was due to reduced postpartum depression or developmental outcomes (Tsivos, Rachel, Sanders, & Anja, 2015). Other reviews have focused on prevention of postpartum depression rather that treatment (Boath, Bradley, & Henshaw, 2004; Dennis, 2005; Morrell et al., 2016).

It has been reported by the World Health Organization (WHO) that the availability of specialized and general health workers dealing with mental health in LMICs is grossly insufficient. This raises concerns and therefore person's with mental health disabilities may not get the treatment required or the service due to a lack of resources (WHO, 2016a). Persons with mental health disabilities may not access services as there the evidence suggests that these are readily available. In view of these factors, the WHO Mental Health Gap Action Program (mhGAP) has recommended the scaling up of services for mental, neurological and substance use disorders, especially in LMICs (WHO, 2016b). Among the psychological therapies, WHO has recommended and also published a manual on group IPT for depression that can be administered by non‐specialists providers in mental health but have some training in IPT (WHO, 2016b).

World Health Organization (WHO, 2016b) recommends that evidence based psychological interventions such as IPT and CBT should be the first line treatment for pregnant and breast‐feeding women with moderate‐severe depression. While CBT can only be delivered by specialized and trained psychologist, individual IPT can be delivered by mental health professionals such as physicians, psychologists, nurses and social health workers. Further, group IPT can be delivered by supervised facilitators who may not have received previous training in mental health (WHO, 2016b). Therefore in line with this idea and goals of WHO's Comprehensive Mental Health Action Plan 2013–2020 (WHO, 2013), IPT can be suggested as the most accessible and affordable therapy among all psychotherapies which can be delivered in general healthcare and community based settings by non‐specialized individuals (WHO, 2016b).

Therefore, this present review will focus on the effectiveness of IPT alone or in combination with pharmacotherapy for treating depressive symptoms. The present review will be useful in generating evidence on the efficacy of IPT in treating postpartum depression. The generated evidence from this review could be used to inform policies in relation to treating postpartum women with depressive symptoms in LMICs.

## 2 OBJECTIVES

### 2.1 Primary objective

a) What is the effectiveness of IPT alone or in combination with pharmacological therapy and/or other psychological and psychosocial interventions in reducing depressive symptoms in women diagnosed with postpartum depression?

### 2.2 Secondary objectives

b) What is the effectiveness of IPT according to the mode, that is, individual, group, conjoint and telephonic; on reducing depressive symptoms in postpartum depression?

c) What is the effectiveness of standard IPT (16 sessions) versus brief IPT (8–12 sessions) on reducing depressive symptoms in postpartum depression?

## 3 METHODS

### 3.1 Criteria for considering studies for this review

#### 3.1.1 Types of studies

We plan to identify and assess experimental, quasi‐experimental and factorial designs where the primary or secondary aim is to find out the effectiveness of IPT for reducing depressive symptoms in women diagnosed with postpartum depression. Study designs such as case reports, case series, reviews and non‐original studies such as editorials, book reviews, commentaries, and letters to editors, will be excluded.

Designs will include:

- Randomized controlled trial in which post‐partum mothers with depression are randomly assigned to intervention and comparison conditions.
  1. Interventions includes Individual or Group Interpersonal psychotherapy
  2. Comparators include usual/standard care or pharmacotherapy or IPT (individual or group) with pharmacotherapy or other psycho‐social interventions or other psycho‐social interventions with pharmacotherapy.

- Quasi‐experimental trials in which the mothers with depression are assigned to groups non‐randomly.
  1. Interventions includes individual or group interpersonal psychotherapy
  2. Comparators include usual/standard care or pharmacotherapy or IPT (individual or group) with pharmacotherapy or other psycho‐social interventions or other psycho‐social interventions with pharmacotherapy.

For randomized and quasi‐experimental trials, studies with significant baseline differences in the primary outcome measures will be excluded.

- Mixed methods where only quantitative components will be included.
- Factorial designs, where IPT and pharmacotherapy is compared to psychosocial interventions along with pharmacotherapy.

Single group designs that measure pre and post outcomes will be excluded.

#### 3.1.2 Types of participants

The specific population of interest is postpartum mothers in LMICs diagnosed with depression developed during pregnancy or within 6 weeks post‐delivery, identified either through clinical diagnosis (DSM‐IV, ICD‐10) or self‐reported measures, for example, Beck Depression Inventory (BDI), EPDS (Edinburg Postnatal Depression Scale), Public Health Questionnaire (PHQ‐9), Hamilton Rating Scale for Depression (HRSD), Postpartum Depression Scale (PPDS), Major Depression Inventory (MDI), Inventory to Diagnose Depression (IDD), Center for Epidemiological Studies Depression Scale (CES‐D) and Zung Self rating depression scale (SDS).

Our review will be international in scope and will apply to LMICs.

#### 3.1.3 Types of interventions

Interventions for the review will include IPT alone or IPT in combination with Pharmacological treatment given to women with postpartum depression.

The studies will be divided into categories to examine the different modes of delivering IPT such as Group‐IPT vs Individual IPT, Brief IPT/IPT‐B (8 weeks) vs IPT (12–16 weeks), Telephonic IPT vs Standard IPT.

The studies will also be sub grouped into categories to examine the effect of IPT intervention delivered by psychiatrist, psychologist, trained nurses/social workers/health visitors and non‐specialized trained volunteers from the community.

##### Treatment comparisons

1. IPT and usual or standard care (includes wait list controls)
2. IPT and pharmacotherapy
3. IPT alone and IPT with pharmacotherapy
4. IPT and combination of psychological and psychosocial interventions
5. IPT and pharmacotherapy compared with psychological and psychosocial interventions with pharmacotherapy

#### 3.1.4 Types of outcome measures

##### Primary outcomes

Authors expect that depressive symptoms such as severity, symptom remission, recovery status and relapse in the primary studies will be measured through clinical diagnosis (DSM or ICD criteria) or self‐reported measures, for example, Beck Depression Inventory (BDI), EPDS (Edinburg Postnatal Depression Scale), Public Health Questionnaire (PHQ‐9), Hamilton Rating Scale for Depression (HRSD), Postpartum Depression Scale (PPDS), Major Depression Inventory (MDI), Inventory to Diagnose Depression (IDD), Centre for Epidemiological Studies Depression Scale (CES‐D) and Zung Self rating depression scale (SDS).

##### Secondary outcomes

- Maternal outcomes: Mother‐infant bonding/attachment, anxiety, social support, postpartum adjustment, social adjustment, transition back to work.
- Couple outcomes: Marital adjustment, sexual interest.
- Adverse effects outcomes: Suicide attempts, suicides, number of hospitalizations, duration of hospitalization, lost workdays, etc.

###### Duration of follow‐up

In order to combat the risk for symptom severity and relapse, acute IPT is never terminated but concluded at the minimum in 8 weeks and maximum in 16 weeks. In untreated cases, the maintenance phase including monthly scheduled sessions may continue afterwards. Hence, the minimum duration of the follow‐up will be 8 weeks. Maximum duration is not limited.

###### Types of settings

All general health settings and community‐based settings will be included. We will also include the study settings where telephone‐based interventions are given.

### 3.2 Search methods for identification of studies

Both published and unpublished studies will be considered eligible for this review. There will be no language restrictions. Relevant studies will be sourced through literature searches of European and American bibliographic databases and general search engines. Gray literature will also be sought. The following databases will be systematically searched:

- Cochrane Central Registry of Controlled Trials.
- PUBMED
- EMBASE
- PsycINFO
- ERIC (ProQuest)
- CINAHL
- Web of Science

The search strategy will firstly develop keywords, combined with Boolean operators, to assess potentially relevant articles on the PubMed database from 1970 until 2019 (see Appendix 2B). IPT was developed in the 1970s, and so we will start our retrieval date from there. Due to controlled vocabularies across each database, the search strategy terms will be adjusted to compensate for different databases. The thesaurus of each database will be checked to ensure no indexed terms are missed. Relevant thesaurus terms will be added to the search strategy. We will report details of the adjustments used for other databases in the completed review. The combination of keywords and thesaurus terms will allow the search strategy to be implemented.

In addition to bibliographic database searches, we will attempt to capture European grey literature through OpenGREY. The authors will contact the experts in the field of IPT to identify the unpublished and ongoing studies. A list of the inclusion criteria for the review will be sent to these experts along with the request for studies. Any studies that match the inclusion criteria will be considered for use in this review.

The reference lists of relevant articles will be hand searched and any potentially relevant studies that are identified will also be retrieved and considered for inclusion. Furthermore, bibliographies and references of systematic reviews and meta‐analysis will also be examined to identify relevant studies in this field. Clinical trials will be sought using the resource ClinicalTrials.gov. This website lists completed and ongoing clinical trials in the US and around the world.

Lastly, we will conduct an advanced Google search where we will input the same keywords used in the bibliographic database search. The first twenty pages of results will be screened. In addition, we will conduct the search again prior to submission to ensure we have collated the most recent data.

#### 3.2.1 Description of methods used in primary research

Anticipated methods that studies are likely to employ include random assignment to treatment/comparison and pre‐test post‐test design. There will be randomized controlled designs where the participants are randomly assigned to intervention or control group and quasi‐experimental designs, where participants are allocated to groups using non‐random methods but steps are taken to control the confounders, for example, matching of groups, measures to ensure baseline equivalence for main outcomes etc. It is expected that treatment condition IPT will be compared to either usual/standard care or pharmacological therapy/other psychosocial therapies.

We will use the secondary outcomes separately in summary of findings. if insufficient data is reported in the primary studies, then narrative account of these outcomes will be provided.

Different studies may use different instruments for measuring the outcomes. We will give careful consideration, whether outcomes from different measures can be combined in meta‐analysis. Further, sensitivity analysis will also be conducted.

### 3.3 Data collection and analysis

#### 3.3.1 Selection of studies

In the first phase, two members of the review team will independently review the titles and abstract to exclude the studies. Full text of only those studies will be retrieved, for which there is agreement between the two reviewers. If disagreement persists regarding exclusion of the studies, a third member of the review committee will be involved to resolve the disagreement.

Second phase will include review of full articles and determine the eligibility based on inclusion criteria, which will be reviewed by two independent members of the review team. Here again, in case of any disagreement, third member of the review team will be consulted.

The rationale for exclusion of the studies will be documented for each full text article reviewed. All the studies will be uploaded in Endnote/Mendeley citation tool. PRISMA diagram will be used to present overall search and screening process.

##### Multiple reports of a single study

Multiple reports by a single study will be considered as one study. If needed, authors of studies will be consulted for clarifying any doubt.

##### Multiple groups in a single study

Data from all the eligible intervention arms will be combined and will be compared with the data from all the eligible control arms. Further single pairwise comparison will be conducted in the meta‐analysis.

If two or more interventions are compared with one control group, then each intervention group will be analyzed separately & sample size will be divided for comparison or control group in order to avoid double counting.

##### Multiple measures of the same outcome

We will calculate mean standardized mean difference (SMD) for all measures of the same outcome variable, with their pooled estimated variance so that only one effect estimate can be contributed by each study for the meta‐analysis.

##### Multiple point in time

For different point in time data, separate analysis will be conducted.

#### 3.3.2 Data extraction and management

##### Details of study coding categories

The studies meeting the inclusion criteria will be reviewed thoroughly and important information will be extracted in tables. Two members of the review team will extract the relevant data from each included study into evidence table. Data abstraction form will be designed to gather pertinent information from included studies such as study population characteristics, setting, intervention, comparators, study design, methods, results, risk of bias, etc. One member of the team will review the data abstraction for completeness and accuracy. In case of any disagreement, discussion will be done to reach the consensus, or another member of the team will be consulted to resolve the disagreement.

Coding categories will be as follows:

1. Publication characteristics:
  • Publication type
  • Author details
  • Date
  • Setting of study (country and place)
2. Participant information
  • Age
  • Country of residence
  • Race/ethnicity
  • Socioeconomic status
3. Intervention
  • Type of intervention
  • Mode of intervention
  • Number of sessions
  • Duration of follow up
4. Comparison
  • Type of comparison
  • Mode
  • Number of sessions
  • Duration of follow up
5. Outcome measures
  • Maternal outcomes
  • Couple outcomes
  • Adverse effect outcomes
  • Tool/instruments used for measurement of each outcome construct
6. Results
7. Risk of bias assessment
  • Random sequence generation
  • Allocation concealment
  • Blinding of participants
  • Blinding of personnel
  • Outcome assessment blinding
  • Attrition bias (incomplete outcome data)
  • Reporting bias (selective reporting)
8. Data analysis
  • Information to compute effect size of each outcome
    (a) Continuous outcome variable: Mean, SD, n for treatment and control group
    (b) Dichotomous outcome variable: number of events and number of events for control and treatment group
  • Effect size
  • Significance level of effect size

#### 3.3.3 Assessment of risk of bias in included studies

For the assessment of risk of bias of included studies of randomized trial designs, revised Cochrane risk of bias tool for randomized tool (RoB 2.0; Higgins et al., 2016) will be used. Risk of bias will be assessed on seven domains; sequence generation, allocation concealment, blinding of participants and personnel, blinding of outcome assessment, incomplete outcome data, selective outcome reporting and other issues. Risk of bias will be judged as low risk, high risk and unclear risk.

For quasi‐experimental studies the ROBINS‐1 tool (Risk of Bias in Non‐randomized Studies‐of Interventions) will be used (Sterne et al., 2016). Studies will be judged as low risk, moderate risk, serious risk and critical risk.

Two trained members of the team will assess the risk of bias of included studies independently. If studies do not report details to assess the validity of design or study, it will be reported as risk of bias to be unclear.

#### 3.3.4 Measures of treatment effect

For each separate comparison, we will perform meta‐analysis for comparison, if two or more similar studies will be found for comparison of variables of interest. RevMan and R software will be used to conduct meta‐analysis, sub‐group analysis, sensitivity analysis, and publication bias estimates.

The included studies will be first categorize based on study designs (i.e., RCT vs quasi‐experimental), and then by intervention type. For multi‐component interventions, we will determine which arms are most relevant to our review objectives and meet our inclusion criteria for meta‐analysis. In a three‐armed RCT ((a) IPT alone, (b) IPT and pharmacotherapy, (c) standard or usual care), first we will include arms 1 and 2 in our analysis, and then 1 and 3 in our analysis. In a two‐armed multi‐component RCT ((a) IPT and pharmacotherapy, (b) no treatment) we will analyze these studies separately from two‐armed single component RCT ((a) IPT alone, (b) standard or usual care).

Descriptions of all intervention arms per study will be presented in “Characteristics of Included Studies” table.

Depending on the outcome, we will calculate the standardized mean difference SMD (for continuous outcomes) or log odds ratio (for dichotomous outcomes), which are appropriate for comparison between groups across similar types of treatment effects.

Where studies report baseline and post‐intervention outcome data, SMDs will be calculated using baseline adjusted mean differences (i.e., mean change scores). Standard deviation for the change score will be standardized using the raw standard deviation within groups. In cases where authors do not report standard deviation for change scores, a formula recommended by Lipsey and Wilson (2001) will be used to estimate these figures.

Where multiple measures are reported for an outcome (e.g., self‐reported depression) in a single study, we will synthesize all measures of depression across all included studies, as this will allow complex analyses.

We will attempt to pool all studies within a given study design and or intervention category, assessing the same outcome, in the same population group by conducting a random‐effects meta‐analysis using Review Manager 5.3. We will generate τ2, or Tau2 to estimate the absolute value of true heterogeneity between studies. Moreover, to ensure no unit‐of‐analysis errors are made, we will combine groups to create a single pair‐wise comparisons for each meta‐analysis. Where meta‐analysis is deemed inappropriate due to substantial heterogeneity, we will summarize the findings of the included studies narratively.

Where some relevant studies have repeated observations on participants, several different outcome measures will be defined on different periods of follow‐up: brief (0–8 weeks), medium (9–16 weeks), and follow‐up (<16 weeks), these different outcomes will be analyzed separately.

A random‐effects meta‐analysis model will be used given the high level of variation in effect sizes anticipated across studies due to the wide diversity in the design, scale, and implementation of their interventions related to IPT and/or their combination. Additionally, heterogeneity will be examined using the I2 statistic, Chi2 test and Tau2 (Deeks, Higgins, & Altman, 2017).

We will calculate pooled effect sizes separately for the posttest comparison of IPT with usual care or no treatment or placebo, with other psychological and psychosocial interventions and pharmacotherapy, combination treatment with IPT and pharmacology compared with pharmacotherapy alone.

We aim to compare treatments; IPT, IPT with pharmacotherapy, IPT and combination of psychological and psychosocial interventions.

#### 3.3.5 Unit of analysis issues

We will asses the unit of analysis errors for cluster randomized trials. If a study is found to be having unit of analysis error, we will use corrected standard error and confidence intervals. Further Intra‐class correlation will also be estimated. Authors of the primary study will be contacted to find out the accuarate measure f intra‐class correlation in order to calculate the corrceted standard error. If authors will not respond then intra‐class correlations will be estimated from the similar types of studies and method of estimation will explained clearly in the review.

#### 3.3.6 Dealing with missing data

The standardized coding form includes information on missing data. In the case that data are missing from documents study authors will be contacted and original data will be sought. Where missing data cannot be recovered, studies will be coded on methods used to cope with missing data and any inherent assumptions made explicit. This data will be included in the final analysis, with the potential impact on findings discussed (Deeks, Higgins, & Altman, 2017).

#### 3.3.7 Assessment of heterogeneity

Heterogeniety is anticipated due to the difference in the type/mode of IPT, study designs and study settings etc. To tackle such type of heterogeneity, we will coduct the seprate anlaysis for diffrent settings, study designs, intervention type/mode etc.

Further, a random‐effects meta‐analysis model will be used given the high level of variation in effect sizes anticipated across studies due to the wide diversity in the design, scale, and implementation of their interventions related to IPT and/or their combination. heterogeneity will be examined using the I^2^ statistic, Chi^2^ test, Q statistics and Tau^2^ (Deeks, Higgins, & Altman, 2017). Heterogeniety will be considered as substatial if I^2^ statistics value is greater than 30% and either T^2^ is greater than zero, or the p value for Chi^2^ test for heterogeniety is <0.10.

#### 3.3.8 Assessment of reporting biases

In order to ascertain whether publication bias may be influencing results a number of gray literature sources will be searched for relevant material. Key authors will be contacted for unpublished documents relevant to the research question. Trial registries will also be searched (e.g., http://www.ctri.nic.in) from a range of countries. A funnel plot will be produced to assess whether publication bias is likely to be influencing overall results and, data permitting, subgroup analyses will be conducted to examine whether intervention effects vary by publication source (published vs unpublished).

#### 3.3.9 Subgroup analysis and investigation of heterogeneity

We will apply the random‐effects model in conducting the subgroup analyses including all between and within subgroup weighting for the specified characteristics below. With this approach, we will estimate and report the variance in effect size across the studies within each subgroup and pool these subgroup estimates based on the assumption that the between study variance is likely to be the same for all subgroups. Combined mean effect estimates of the subgroups will be computed based on the random‐effects weights and pooled tau2. Comparisons for statistically significant differences will be tested using an analysis of variance Q statistic that follows a chi‐square distribution.

We have identified subgroup analyses that would be most appropriate to investigate heterogeneity (if data permits):

- Different modes of delivering IPT (Group‐IPT vs Individual IPT, Brief IPT/IPT‐B [8 weeks] vs IPT [12–16 weeks], Telephonic IPT vs Standard IPT)
- Effect of IPT intervention delivered by psychiatrist, psychologist, trained nurses/social workers/health visitors and non‐specialized trained volunteers from community
- Effect of IPT as per setting, for example, general health settings, community‐based setting, telephone‐based interventions

For both categorical and continuous variables, meta‐regression will be used. However, it can be prone to overfitting when the number of studies included in the analysis is quite small (van Houwelingen, Arends, & Stijnen, 2002). Where there is insufficient data to conduct meta‐regression, moderator analysis analogous to ANOVA will be used for categorical factors.

#### 3.3.10 Sensitivity analysis

Where possible, sensitivity analyses will be conducted to examine a range of factors that may impact the robustness of the meta‐analysis results.

These factors include:

(a) Research designs (RCT or quasi‐RCT)
(b) Risk of bias (low, medium or high)
(c) Comparison conditions (e.g., different groups of comparison conditions)
(d) Timing of the outcome (e.g., 8 weeks, 16 weeks)

Any additional issues identified during the review process will also be analyzed. Where certain decisions are identified to have potential influence on findings, sensitivity analysis will be undertaken by performing analysis with certain subgroups removed. The results of sensitivity analysis will be presented in a summary table.

If due to any reason such as heterogeneity, very few similar studies or outcome measures variability; each study findings will be described separately.

##### Summary of findings and assessment of the certainty of the evidence

The summary of findings will be presented in the table based on GRADE recommendations. The table will include outcome variables, Quality of evidence, sample size of interventional and control group, effect size and results.

## CONTRIBUTIONS OF AUTHORS

### Roles and responsibilities

- Content: Bandana Bisht, Harmeet Kaur.
- Systematic review methods: Harmeet Kaur, Obrey Alexis, Denny John, Bandana Bisht, Manmeet Kaur.
- Statistical analysis: Denny John, Harmeet Kaur.
- Information retrieval: Denny John, Aaron Worsley.

## DECLARATIONS OF INTEREST

No potential conflicts involved. Bandana Bisht is a trained IPT therapist. At present, she is not conducting any primary research in IPT for postpartum depression.

## APPENDIX A AUTHORS' DECLARATION

### Authors’ responsibilities

By completing this form, you accept responsibility for preparing, maintaining and updating the review in accordance with Campbell Collaboration policy. Campbell will provide as much support as possible to assist with the preparation of the review.

A draft review must be submitted to the relevant Coordinating Group within two years of protocol publication. If drafts are not submitted before the agreed deadlines, or if we are unable to contact you for an extended period, the relevant Coordinating Group has the right to de‐register the title or transfer the title to alternative authors. The Coordinating Group also has the right to de‐register or transfer the title if it does not meet the standards of the Coordinating Group and/or Campbell.

You accept responsibility for maintaining the review in light of new evidence, comments and criticisms, and other developments, and updating the review at least once every five years, or, if requested, transferring responsibility for maintaining the review to others as agreed with the Coordinating Group.

### Publication in the Campbell Library

The support of the Coordinating Group in preparing your review is conditional upon your agreement to publish the protocol, finished review, and subsequent updates in the Campbell Library. Campbell places no restrictions on publication of the findings of a Campbell systematic review in a more abbreviated form as a journal article either before or after the publication of the monograph version in Campbell Systematic Reviews. Some journals, however, have restrictions that preclude publication of findings that have been, or will be, reported elsewhere and authors considering publication in such a journal should be aware of possible conflict with publication of the monograph version in Campbell Systematic Reviews. Publication in a journal after publication or in press status in Campbell Systematic Reviews should acknowledge the Campbell version and include a citation to it. Note that systematic reviews published in Campbell Systematic Reviews and co‐registered with Cochrane may have additional requirements or restrictions for co‐publication. Review authors accept responsibility for meeting any co‐publication requirements.

**I understand the commitment required to undertake a Campbell review, and agree to publish in the Campbell Library. Signed on behalf of the authors**:

**Table:**

**Form completed by: Harmeet Kaur Kang**	**Date: 06.14.2019**

## APPENDIX B INITIAL SEARCH TERMS

There will be two categories of keywords employed for this search. The first category of terms will list keywords related to the condition of interest—postpartum depression. Our review is only concerned with postpartum depression, and use of pnd as a search term will find articles relating to both postnatal depression and perinatal depression. Articles concerning perinatal depression will be discarded. The second category of terms will relate to the intervention—IPT. The third category, outcomes, has been omitted due to the limitation this will place on sourcing relevant articles. Including keywords relating to effectiveness will exclude studies that may incorporate other indexed terms.

The search terms will be linked by use of the Boolean operator AND, appearing as so:

**Condition of Interest:** “postpartum depressi*” OR “post‐partum depressi*” OR “postnatal depressi*” OR “post‐natal depressi*” OR ppd OR pnd

**Intervention:** psychotherap* OR therap* OR counsel*

The final search terms, including thesaurus terms, will be detailed in the full review.[Bibr cl21074-bib-0075]
